# Efficient genomic prediction based on whole-genome sequence data using split-and-merge Bayesian variable selection

**DOI:** 10.1186/s12711-016-0225-x

**Published:** 2016-06-29

**Authors:** Mario P. L. Calus, Aniek C. Bouwman, Chris Schrooten, Roel F. Veerkamp

**Affiliations:** Animal Breeding and Genomics Centre, Wageningen UR Livestock Research, PO Box 338, 6700 AH Wageningen, The Netherlands; CRV BV, 6800 AL Arnhem, The Netherlands

## Abstract

**Background:**

Use of whole-genome sequence data is expected to increase persistency of genomic prediction across generations and breeds but affects model performance and requires increased computing time. In this study, we investigated whether the split-and-merge Bayesian stochastic search variable selection (BSSVS) model could overcome these issues. BSSVS is performed first on subsets of sequence-based variants and then on a merged dataset containing variants selected in the first step.

**Results:**

We used a dataset that included 4,154,064 variants after editing and de-regressed proofs for 3415 reference and 2138 validation bulls for somatic cell score, protein yield and interval first to last insemination. In the first step, BSSVS was performed on 106 subsets each containing ~39,189 variants. In the second step, 1060 up to 472,492 variants, selected from the first step, were included to estimate the accuracy of genomic prediction. Accuracies were at best equal to those achieved with the commonly used Bovine 50k-SNP chip, although the number of variants within a few well-known quantitative trait loci regions was considerably enriched. When variant selection and the final genomic prediction were performed on the same data, predictions were biased. Predictions computed as the average of the predictions computed for each subset achieved the highest accuracies, i.e. 0.5 to 1.1 % higher than the accuracies obtained with the 50k-SNP chip, and yielded the least biased predictions. Finally, the accuracy of genomic predictions obtained when all sequence-based variants were included was similar or up to 1.4 % lower compared to that based on the average predictions across the subsets. By applying parallelization, the split-and-merge procedure was completed in 5 days, while the standard analysis including all sequence-based variants took more than three months.

**Conclusions:**

The split-and-merge approach splits one large computational task into many much smaller ones, which allows the use of parallel processing and thus efficient genomic prediction based on whole-genome sequence data. The split-and-merge approach did not improve prediction accuracy, probably because we used data on a single breed for which relationships between individuals were high. Nevertheless, the split-and-merge approach may have potential for applications on data from multiple breeds.

**Electronic supplementary material:**

The online version of this article (doi:10.1186/s12711-016-0225-x) contains supplementary material, which is available to authorized users.

## Background

Genomic selection was introduced in many livestock breeding programs during the last decade. One of the main reasons for its rapid development is the availability of 50k single nucleotide polymorphism (SNP) chips for all major livestock species, including cattle [1], pigs [2], and poultry [3]. Following earlier predictions that a higher SNP density was necessary to enhance persistence of genomic predictions across generations [4–6] and breeds [7–9], SNP chips with at least ten times more SNPs were developed for cattle [10], pigs (personal communication MAM Groenen and AL Archibald), and poultry [11]. The added benefit of using these higher-density SNP chips for genomic selection is at best limited, both across generations (e.g. [12]) and across breeds [9].

At the same time, simulation studies suggested, rather optimistically, that the use of (imputed) whole-genome sequence data could result in considerably increased accuracy of genomic prediction [5, 6]. An important limitation for genomic prediction using whole-genome sequence data, is the required computation time due to the sharp increase in SNP number, e.g. from 50k to over 10 million. One strategy to deal with this, involves pre-selection of SNPs that can, for instance, be based on the data from genome-wide association studies using imputed sequence data, and then on the selection of the SNPs with a significant association. Brøndum et al. [13] showed that combining data from such SNPs with the 50k-SNP chip data can slightly increase the reliability of genomic prediction by up to 5 percentage points, but Veerkamp et al. [14] found no improvement in reliability using a similar approach. However, the initial idea behind genomic prediction was to simply include all SNPs in the genomic prediction model, and to perform variable selection [15]. Early results from empirical studies with imputed sequence data that simply included all imputed SNPs together in one genomic prediction model, showed little or no improvement over the use of 50k or higher-density SNP chips, even when a Bayesian variable selection model was used, which is expected to identify the SNPs associated with the trait of interest [16, 17]. There are several possible explanations for this result: (1) imputed sequence data may still miss a significant proportion of the causal SNPs; (2) strong LD between multiple SNPs and a QTL that potentially segregate together in long haplotypes, makes it more difficult to pinpoint the causal SNP, even when it is included in the data; and (3) the *n* ≪ *p* (*n* for number of recorded phenotypes and *p* for number of estimated SNP effects) problem is much greater with imputed sequence data than with e.g. the 50k-SNP chip, because *p* increases considerably, while *n* remains the same. In addition, computation time increases proportionally to the sharp increase in *p*.

The issues involved in genomic prediction using sequence data, as described above, could be, at least partly, solved by reducing the number of SNPs by removing those that are not associated with the trait of interest. This can be achieved by performing a genome-wide association study [13, 18], or by pooling SNPs based on functional data [17]. An alternative is to first apply a Bayesian variable selection model independently to subsets of the sequence data, such that SNPs are selected within each of the subsets. The second step involves merging of the selected SNPs across the subsets, and then analyzing the merged dataset by using a variable selection model to estimate the final genomic predictions. This approach was proposed for ultrahigh-dimensional regression, of which genomic prediction using whole-genome sequence is one example, and is termed “split-and-merge Bayesian variable selection” [19]. To date and to our knowledge, no applications of split-and-merge (SAM) Bayesian variable selection for genomic prediction using sequence data have been reported.

The objective of this study was to investigate the accuracy of SAM Bayesian variable selection applied to whole-genome sequence data. SAM modelling involves a series of initial analyses on subsets of SNPs, to select a final set of SNPs that are in strong association with the trait of interest. Our hypothesis is that the SAM Bayesian variable selection model is able to alleviate the severe *n* ≪ *p* problem that is encountered when all SNPs in the sequence data are simultaneously fitted in a single genomic prediction model.

## Methods

### Data

The data included de-regressed proofs (DRP) and effective daughter contributions (EDC; [20]) for 5556 Holstein–Friesian bulls for somatic cell score (SCS), protein yield (PY), and interval first to last insemination (IFL) that were available from CRV (Cooperative Cattle Improvement Organization, Arnhem, The Netherlands). In addition, the bulls were genotyped with the Illumina BovineHD Bead chip (HD; 734,403 SNPs; Illumina Inc., San Diego), or with the 50k-SNP panel and the genotypes were then imputed to high-density.

### Imputation to whole-genome sequence

The BovineHD genotypes of the bulls were imputed to whole-genome sequence using the sequenced individuals from the 1000 Bull Genomes Project Run 4 as reference population. This multi-breed reference population comprised 1147 sequenced animals with on average an 11-fold coverage of which 311 were Holstein bulls, but all the individuals were included in the reference population because earlier studies showed that a multi-breed sequenced reference population can be beneficial for imputation accuracy, especially for variants with a low minor allele frequency (MAF) [21–23]. Polymorphic sites, including SNPs and short insertions and deletions, were identified across the 1147 individuals using the multi-sample approach implemented in SAMtools’ mpileup along with the BCFtools as described in Daetwyler et al. [23]. Since the considered polymorphic sites include different types of variants, they will hereafter be jointly referred to as (sequence-based) variants. The genotype calls of the 1000 Bull Genomes reference population were improved with BEAGLE [24] using genotype likelihoods from SAMtools and inferred haplotypes in the samples. The sequence data contained 36,916,855 variants of which 30,339,468 with four or more copies in the reference population were used for imputation. HD genotypes were imputed to whole-genome sequence by using standard settings in MINIMAC2 [25] and the pre-phased reference genotypes obtained from BEAGLE. Selection of variants that had a MAF higher than 0.01 in the imputed Holstein population reduced the number of variants to 13,968,906.

Editing steps of the imputed sequence data involved removing variants for which all three genotypes were not observed and thus reduced their number from 13,968,906 to 12,254,506 variants (Table 1).

**Table 1:** Overview of the number of variants removed during each of the editing steps

Description	Number of variants	
	Total	Removed
Initial total	13,968,906	
Only heterozygotes		179,871
Only two genotypes observed		1,534,529
Complete LD with other variants		8,004,933
High LD within subsets		95,509
Finally remaining	4,154,064	

### Strategy to split genotyping data

Initially, the strategy to split the genotyping data was based on creating subsets of equally-spaced variants in terms of their relative position on the genome. Considering that *n* subsets would be formed, the subset *x* would contain variant *x*, *n* + *x*, 2*n* + *x*, etc. However, a preliminary analysis within those subsets showed that several subsets contained quite a few variants that displayed very high levels of linkage disequilibrium (LD) between them, which reduced model performance as observed from the strongly inflated SNP variance component. To alleviate this problem, the following additional edits were performed. Throughout the whole genome, for any group of variants with a squared correlation of 1 between genotypes in the reference population, only the “rightmost” variant was retained, which drastically reduced the number of variants from 12,254,506 to 4,249,573 (Table 1).

Starting with these 4,249,573 variants, to further reduce the levels of LD within the subsets, subsets were formed using an alternative strategy. Variants were first sorted based on their MAF, which was used as a very simple proxy for high LD, considering that high LD between two loci can only be achieved if the loci have similar MAF [26, 27]. Based on the list of variants sorted in this way, 106 (= *n*) subsets of 40,090 or 40,091 variants were formed, where subset *x* contained variant *x*, *n* + *x*, 2*n* + *x*, etc. The first 33 subsets contained 40,091 variants and the last 73 subsets contained 40,090 variants.

Initial analyses on these subsets revealed that, in spite of sorting variants on MAF to allocate those in high LD across subsets, the performance of the model for several subsets still suffered from levels of LD that were too high. To alleviate this, for all subsets, the variant groups that had a squared correlation between genotypes higher than 0.95, only the “rightmost” was retained. For a limited number of subsets, the performance of the model was still poor, thus we repeated the same editing step again with a lower threshold of 0.90 for 16 subsets, and again with a threshold of 0.85 for two of those 16 subsets. Finally, the combined sorting of variants based on MAF and checking for LD within subsets reduced the number of variants from 4,249,573 to 4,154,064. See Table 1 for an overview of the number of variants removed during each editing step.

### Model

Within each of the 106 subsets, a Bayesian stochastic search variable selection model (BSSVS) was applied for each of the three traits. The BSSVS model can be described as:

$${\mathbf{y}} = \mathbf{1}\mu + {\mathbf{Zu}} + {\mathbf{X}}\varvec{\alpha} + {\mathbf{e}},$$

where $1$ is a vector of ones, $\mu$ is the overall mean, ${\mathbf{u}}$ is a vector that contains residual polygenic effects of all bulls distributed as ${\mathbf{u}}\left| {{\mathbf{A}},\sigma_{u}^{2} } \right. \sim N\left( {\mathbf{0},{\mathbf{A}}\sigma_{u}^{2} } \right)$, where ${\mathbf{A}}$ is the numerator relationship matrix derived from the pedigree and $\sigma_{u}^{2}$ is the polygenic variance, ${\mathbf{X}}$ is a matrix that contains centered and scaled genotypes where each column $j$, denoted as vector ${\mathbf{x}}_{j}$, contains the genotypes of locus $j$ (which takes the values of $\frac{{0 - 2p_{j} }}{{\sqrt {2p_{j} \left( {1 - p_{j} } \right)} }}$, $\frac{{1 - 2p_{j} }}{{\sqrt {2p_{j} \left( {1 - p_{j} } \right)} }}$, or $\frac{{2 - 2p_{j} }}{{\sqrt {2p_{j} \left( {1 - p_{j} } \right)} }}$), for all variants (columns) for all bulls (rows), with $p_{j}$ the allele frequency at locus $j$, ${\varvec{\upalpha}}$ is a vector that contains the (random) effects for all variants, and ${\mathbf{e}}$ is a vector of residuals distributed as ${\mathbf{e}}\left| {{\mathbf{D}},\sigma_{e}^{2} } \right. \sim N\left( {\mathbf{0},{\mathbf{D}}\sigma_{e}^{2} } \right)$, where ${\mathbf{D}}$ is a diagonal matrix containing e.g. $1/EDC_{i}$ on the diagonal $i$, where $EDC_{i}$ is the EDC value of bull $i$, and $\sigma_{e}^{2}$ is the residual variance. The prior for $\upmu$ was a constant and both $\sigma_{u}^{2}$ and $\sigma_{e}^{2}$ had a flat, uninformative prior distribution.

In BSSVS, the effect for locus $j$, $\alpha_{j}$, is sampled at each iteration from its conditional posterior density:

$$N\left( {\hat{\alpha }_{j} ; \frac{{\upomega_{j} \hat{\sigma }_{e}^{2} }}{{{\mathbf{x}}_{j}^{{\prime }} {\mathbf{D}}^{ - 1} {\mathbf{x}}_{j} + \lambda_{j} }}} \right),$$

where $\lambda_{j} = \frac{{\upomega_{j} \hat{\sigma }_{e}^{2} }}{{\hat{\sigma }_{\alpha }^{2} }},$ which includes $\upsigma_{\alpha }^{2}$ hereafter referred to as the SNP variance component, and $\hat{\alpha }_{j}$ is the conditional mean of the effect at locus $j$, which is computed as:

$$\hat{\alpha }_{j} = \frac{{{\mathbf{x}}_{j}^{{\prime }} {\mathbf{D}}^{ - 1} {\mathbf{y}}_{j}^{*} }}{{{\mathbf{x}}_{j}^{{\prime }} {\mathbf{D}}^{ - 1} {\mathbf{x}}_{j} +\uplambda_{j} }},$$

where ${\mathbf{y}}_{j}^{*}$ is a vector of conditional phenotypes for locus $j$, defined as ${\mathbf{y}}_{j}^{*} = \mathbf{y} - \mathbf{1}\hat{\mu } - {\mathbf{Z}}\hat{\mathbf{u}} - {\mathbf{X}}_{1:j - 1} {\hat{\varvec{\alpha }}}_{1:j - 1} - {\mathbf{X}}_{j + 1:n} {\hat{\varvec{\alpha }}}_{j + 1:n}$, i.e. the phenotypes corrected for all estimated effects other than those of locus $j$, $n$ is the total number of variants in the analysis, and

$$\begin{array}{*{20}l} {\upomega_{j} = 1} \hfill &\quad {\text{if}} \hfill & {I_{j} = 1,} \hfill \\ {\upomega_{j} = 100} \hfill &\quad {\text{if}} \hfill & {I_{j} = 0.} \hfill \\ \end{array}$$

The conditional posterior density of $\upsigma_{\alpha }^{2}$ is an inverse-$\upchi^{2}$ distribution:

$$\upsigma_{\alpha }^{2} |{\varvec{\upalpha}}\sim\upchi^{ - 2} \left( {\nu_{\alpha } + n, {\text{S}}_{\alpha }^{2} + {\varvec{\upomega}}^{{\prime }} {\hat{\varvec{\alpha }}}^{2} } \right),$$

where $\nu_{\alpha }$ is the number of degrees of freedom that was set to 4.2 following [28], and the scale parameter $S_{\alpha }^{2}$ is calculated as $S_{\alpha }^{2} = \frac{{\tilde{\sigma }_{\alpha }^{2} \left( {\nu_{\alpha} - 2} \right)}}{\nu_{\alpha} }$, ${\hat{\varvec{\alpha }}}^{2}$ is a vector of the squares of the current estimates of the effects of all loci, weighted by vector ${\varvec{\upomega}}$, which contains values $\upomega_{j}$ of 1 or 100 for each locus $j$.

Finally, the conditional posterior distribution of the indicator $I_{j}$ for being associated with a quantitative trait locus (QTL) was:

$$\Pr \left( {I_{j} = 1} \right) = \frac{{{\text{f}}(r_{j} |I_{j} = 1)\left( {1 - \pi } \right)}}{{{\text{f}}\left( {r_{j} |I_{j} = 0} \right)\pi + {\text{f}}(r_{j} |I_{j} = 1)\left( {1 - \pi } \right)}},$$

where $1 - \pi$ ($\pi$) is the prior probability that $I_{j} = 1$ ($I_{j} = 0$), $r_{j} = {\mathbf{x}}_{j}^{{\prime }} {\mathbf{D}}^{ - 1} {\mathbf{y}}_{j}^{*} + {\mathbf{x}}_{j}^{{\prime }} {\mathbf{D}}^{ - 1} {\mathbf{x}}_{j} \hat{\alpha }_{j}$, where ${\mathbf{y}}_{j}^{*}$ contains the conditional phenotypes as defined previously, and $f(r_{j} |I_{j} =\updelta)$, where $\updelta$ is either 0 or 1, is proportional to $\frac{1}{\sqrt v }{\text{e}}^{{ - \frac{{r_{j}^{2} }}{2v}}}$, where $v = \left( {{\mathbf{x}}_{j}^{{\prime }} {\mathbf{D}}^{ - 1} {\mathbf{x}}_{j} } \right)^{2} \frac{{\upsigma_{{\alpha_{j} }}^{2} }}{{\upomega_{j} }} + {\mathbf{x}}_{j}^{{\prime }} {\mathbf{D}}^{ - 1} {\mathbf{x}}_{j}\upsigma_{e}^{2}$.

The conditional posterior densities of $\upsigma_{u}^{2}$ and $\upsigma_{e}^{2}$ were inverse-$\upchi^{2}$ distributions, respectively $\upsigma_{u}^{2} |{\mathbf{u}},{\mathbf{A}}^{ - 1} \sim\upchi^{ - 2} \left( {n_{p} - 2,{\mathbf{u}}^{{\prime }} {\mathbf{A}}^{ - 1} {\mathbf{u}}} \right)$ and $\upsigma_{e}^{2} |{\mathbf{e}},{\mathbf{D}}^{ - 1} \sim\upchi^{ - 2} \left( {n_{r} - 2,{\mathbf{e}}^{{\prime }} {\mathbf{D}}^{ - 1} {\mathbf{e}}} \right)$, where $n_{p}$ is the number of animals in the pedigree, and $n_{r}$ is the number of animals with records.

The Gibbs sampler was implemented using right-hand-side updating [29]. For each of the subsets, a Gibbs chain of 30,000 with a burn-in of 10,000 iterations was run. More information on the implementation of this model is in [29]. For all subsets, the parameter $\pi$ was set to 0.999.

### Merge (selected) genotyping data

Based on the results of the analyses within subsets, variants were sorted based on their posterior probabilities, i.e. the posterior means of the QTL-indicators $I_{j}$. Then, genotypes of the top ranked variants were merged as follows. In the merged data file, variants 1 to 106 were the number 1 ranked variants of subsets 1 to 106, variants 107 to 212 were the number 2 ranked variants of subsets 1 to 106, etc. From each subset, the 5000 variants with the highest posterior probability were included in the merged genotype file, yielding a total of 530,000 variants. Again to avoid problems due to high levels of LD between variants, in this merged dataset, of pairs of variants that had a squared correlation between the genotypes higher than 0.99, only the “rightmost” variant was retained, which reduced the number of variants in the merged dataset from 530,000 to 460,158 for SCS, 471,528 for IFL, and 472,492 for PY. Subsequently, the BSSVS model was run using the first 1060, 5300, 10,600, 53,000, 106,000 variants for each of the traits, or all the variants (460,158, 471,528, or 472,492) in the merged genotype dataset.

### Alternatives to the split-and-merge strategy

To compare the SAM procedure based on imputed sequence data to other strategies, all predictions with the merged subsets were either performed with or without adding all 50k SNPs from the BovineSNP50 chip (Illumina Inc., San Diego). In total, 41,682 SNPs on the commonly used 50k-SNP chip were included in the dataset after imputation to whole-genome sequence, and were used in all the scenarios that included the SNPs of the 50k-chip. Predictions were also performed using only the 50k SNPs or all 4,154,064 variants that included the 41,682 SNPs of the 50k-chip. As a final alternative, genomic estimated breeding values (GEBV) for the selection candidates were computed as the average of the GEBV obtained within each of the 106 subsets. This can be considered as an average of the predictions generated from randomly well-spaced and informative genome-wide 50k-like sets of variants.

Because some of the analyses on the merged datasets and some of the alternative strategies described above involved considerably more variants than the analyses for the subsets, all analyses on the merged datasets and the alternative strategies were performed by running a Gibbs chain of 300,000 iterations with a burn-in of 50,000. For all merged datasets, the parameter $\pi$ was set to 0.999, thus, analyzing all 4,154,064 variants in a single analysis, implied that 4154 of the variants were assumed to have a large effect. To investigate the impact of the value used for $\pi$, a limited number of the merged datasets was also analyzed with a value of $\pi = \frac{{n_{merged} - 4154}}{{n_{merged} }}$ where $n_{merged}$ is the number of variants included in the merged dataset.

### Model convergence

One potential benefit of the SAM BSSVS approach, as indicated above, is that within the subsets the *n* ≪ *p* problem is expected to be much less severe than for an analysis that involves all the variants, simply because the number of variants (*p*) within each subset is much smaller. In addition, both the creation of subsets of variants and the editing carried out to remove variants in complete or high LD, are expected to have a positive impact on model performance within the subsets, in terms of convergence. To assess model convergence, effective sample sizes of the SNP variance component $\upsigma_{\alpha }^{2}$ of all the analyses were computed following [30]. In addition, for the analyses on the merged subset, and those including all variants, the correlation was computed between the posterior GEBV obtained after 50,000 versus 250,000 iterations of the same Gibbs chain after the burn-in. This correlation is an indication of the change of the GEBV between 50,000 and 250,000 iterations after the burn-in.

### Evaluation of predictions

To evaluate prediction accuracy of all applied models, the animal data were split into 3415 reference bulls, all born before 2001, and 2138 validation bulls born between 2001 and 2008. For all the models described previously, only the phenotypes of the 3415 reference bulls were used. The targeted prediction for validation bull $i$, i.e. its GEBV, was computed as $GEBV_{i} = u_{i} + {\mathbf{x}}_{{\mathbf{i}}}^{{\prime }} {\varvec{\upalpha}}$, where ${\mathbf{x}}_{{\mathbf{i}}}^{{\prime }}$ contains the genotypes of bull $i$. Prediction accuracies were computed as the correlation between the DRP and the computed GEBV for the 2138 validation bulls. Bias was assessed via the coefficient of the regression of the DRP on computed GEBV for the validation bulls.

To investigate the impact of using the different final datasets on the detection of QTL, which is the ultimate goal when using sequence data for genomic prediction, the posterior probabilities of all the variants of all models were evaluated. This was done by displaying the Manhattan plots with Bayes factors greater than 1. Bayes factors were computed as:

$$BF = \frac{{Pr\left( {H_{1} |y} \right)}}{{1 - Pr\left( {H_{1} |y} \right)}} \div \frac{{Pr\left( {H_{1} } \right)}}{{1 - Pr\left( {H_{1} } \right)}},$$

where $H_{1}$ is the hypothesis that the variant has a large effect, $Pr\left( {H_{1} |y} \right)$ is the posterior probability of the hypothesis and $Pr\left( {H_{1} } \right)$ is the prior probability of the hypothesis. $1 - Pr\left( {H_{1} |y} \right)$ and $1 - Pr\left( {H_{1} } \right)$ represent, the posterior and prior probability for the alternative hypothesis, respectively. A high Bayes factor indicates that a variant is strongly associated with the trait.

## Results

### Genomic prediction in the split subsets

**Fig. 1:**
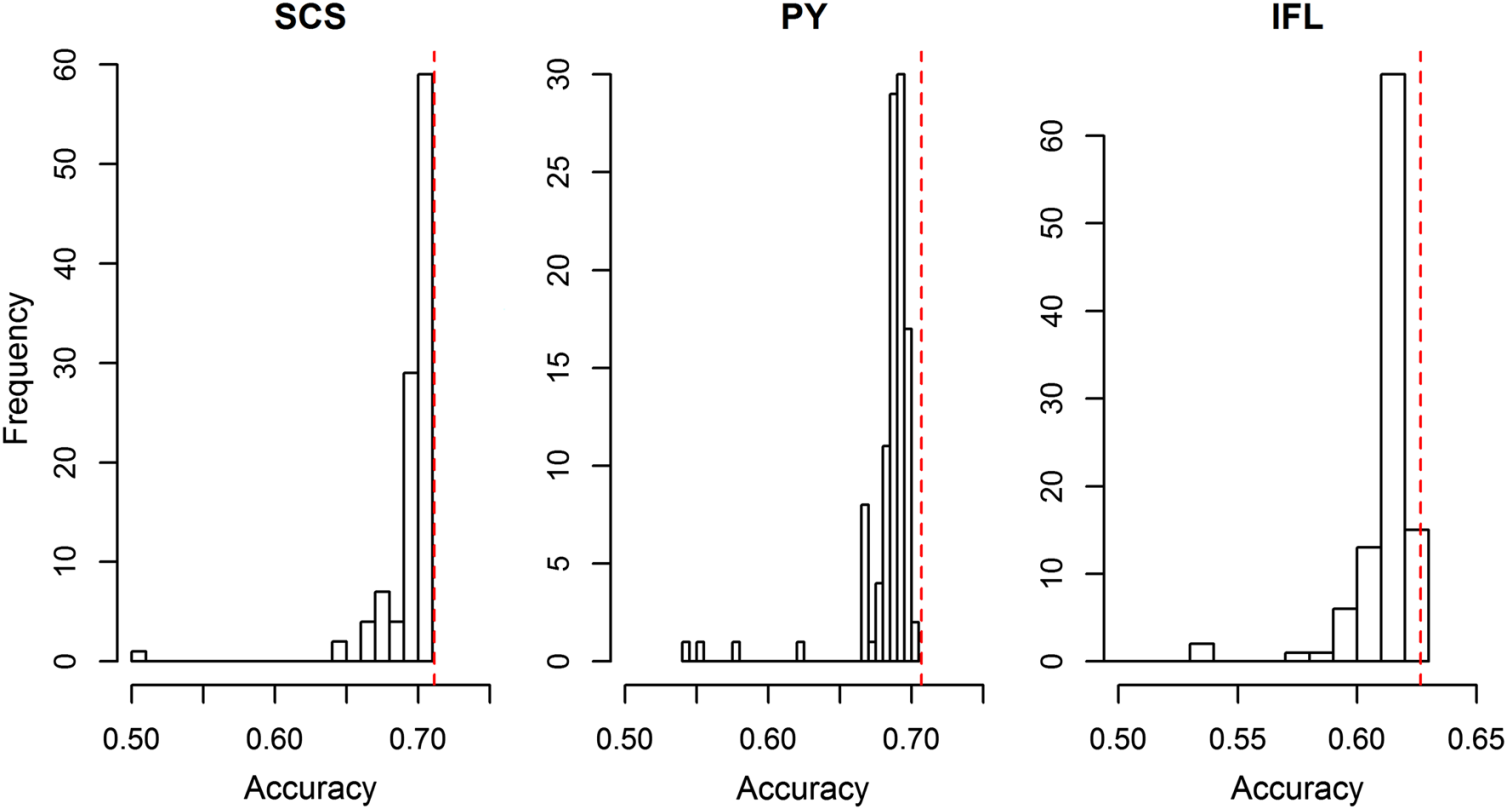
Prediction accuracies achieved in each of the 106 subsets for SCS, PY and IFL. The *dashed red vertical line* indicates the prediction accuracy obtained using 41,682 SNPs included on the 50k-SNP chip

**Fig. 2:**
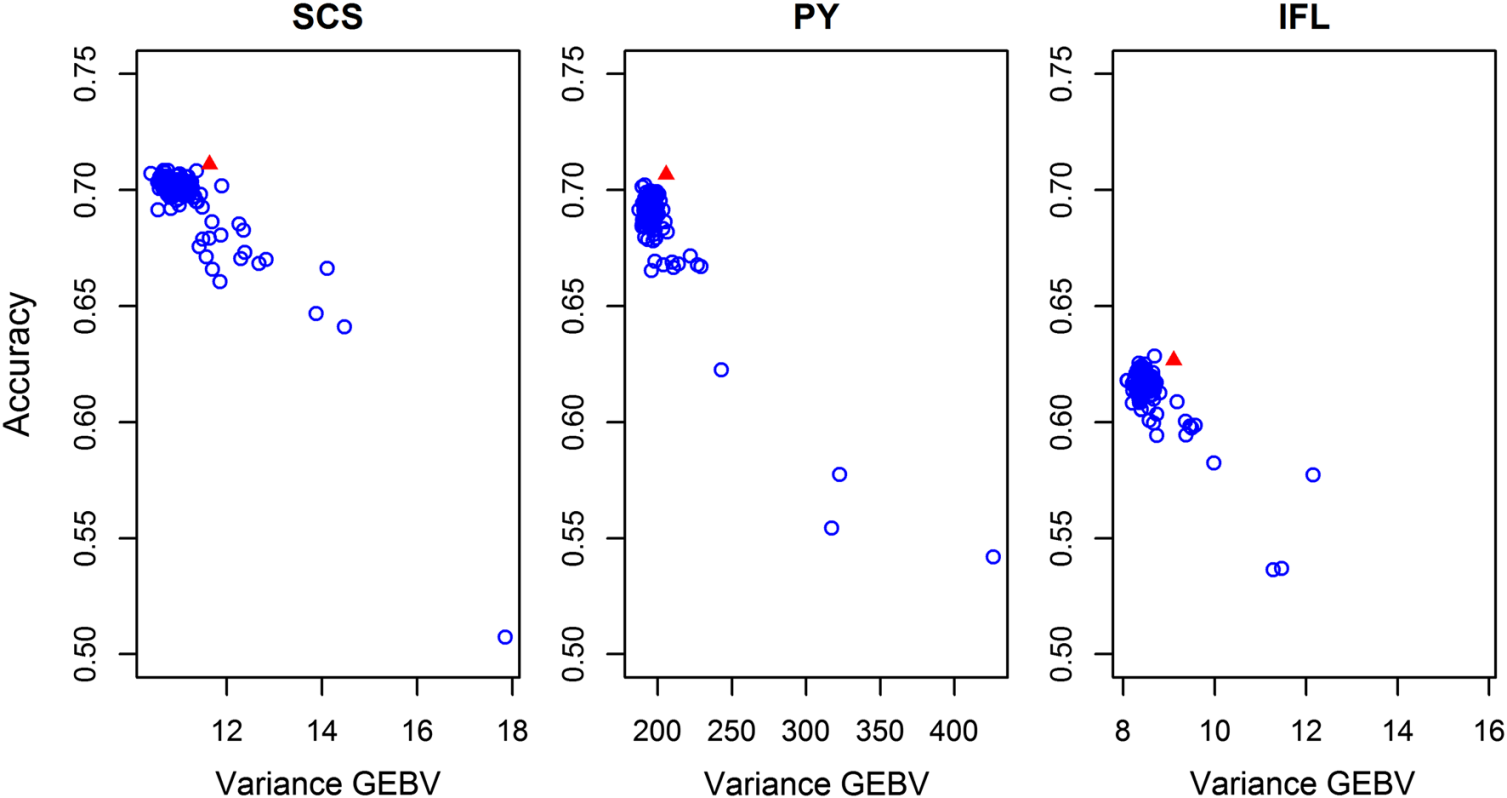
Prediction accuracies versus variance of GEBV in each of the 106 subsets for SCS, PY and IFL. *Open blue circles* indicate values for subsets. The *red solid triangle* indicates the prediction accuracy and variance of GEBV obtained using SNPs included on the 50k-SNP chip

For each of the 106 subsets, the estimated effects of variants were used to predict GEBV for the 2138 validation animals. These prediction accuracies were generally quite close to the accuracies obtained with the 50k-SNP chip, although some subsets yielded slightly lower accuracies, and a few subsets even considerably lower accuracies (Fig. 1). Plotting the prediction accuracies against the variance of the GEBV revealed that prediction accuracies that were similar to those obtained with the 50k-SNP chip were associated with a slightly smaller variance of the GEBV than that with the 50k-SNP chip (Fig. 2). In addition, most of the lower prediction accuracies obtained for subsets were associated with an inflated variance of the GEBV. This suggests that for some of the subsets, analyses still suffered from the co-linearity being too high among the variants included, in spite of the edits performed to alleviate this issue.

To further illustrate the poor model performance due to high LD, accuracies for SCS obtained before the additional step of LD pruning were plotted against the variance of the GEBV for the 16 subsets for which additional LD pruning was performed (see Additional file 1: Figure S1A). This figure clearly shows that, in some cases, the variance of the GEBV was severely inflated and associated with considerably decreased accuracies. Comparison of the initial accuracies against those after the additional LD pruning step showed that, in most cases, this step largely resolved the issue.

### Genomic prediction using the merged final dataset

**Fig. 3:**
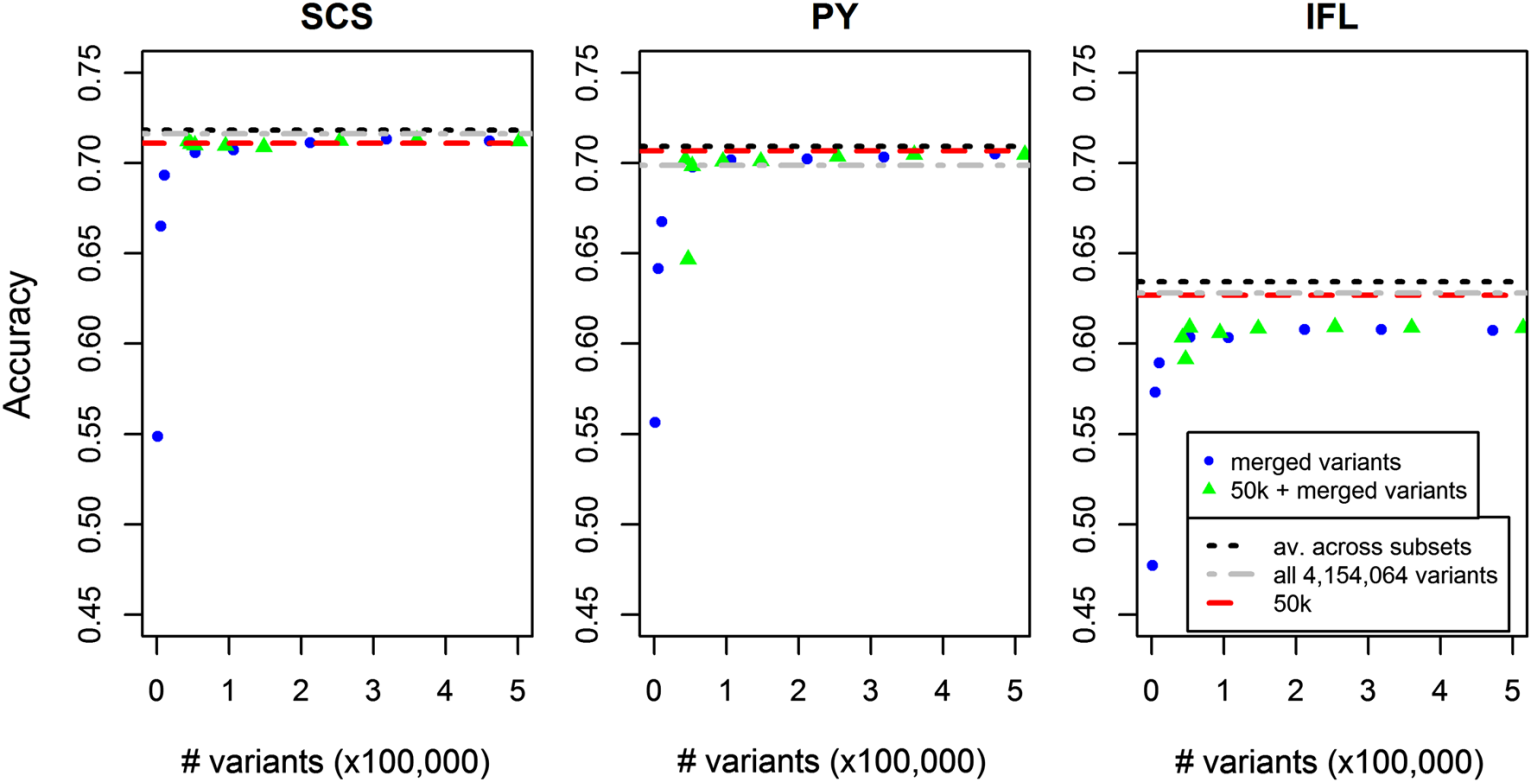
Prediction accuracies achieved using increasingly more variants in the merged dataset for SCS, PY and IFL. Merged variants were either included alone (*blue dots*) or combined with the 41,682 50k-chip SNPs (*green triangles*). *Horizontal lines* indicate the prediction accuracy obtained using the average GEBV across subsets (*dashed black*), GEBV computed using all 4,154,064 variants (*dot-dashed grey*), or the 50k SNPs (*red dashed*)

Using the merged dataset, after selecting variants from the subsets based on posterior probabilities, analyses were performed using a minimum of 1060 variants and a maximum of 460,158 variants for SCS, 471,528 variants for PY, and 472,492 variants for IFL. Accuracies increased considerably as the number of variants included increased up to ~53,000 variants, while small additional increases were observed as the number of variants increased until the maximum values (Fig. 3). When the 50k SNPs were added to the merged dataset, accuracies started to increase at a higher level when the number of added variants from the merged dataset was small, but they reached values very close to those based on the merged data without the 50k SNPs as the number of variants used increased (Fig. 3). In almost all cases, the maximum accuracies that were achieved using the variants from the merged dataset were at best equal to those achieved using only the 50k SNPs. In fact, for IFL, using only the 50k SNPs resulted in considerably better accuracy than using any number of variants from the merged dataset, regardless of whether the 50k SNPs were included or not. The accuracies of the GEBV that were computed as the average of the GEBV computed for each of the 106 subsets, were highest for all three traits and were 0.5 to 1.1 % higher than those obtained with the 50k SNPs. Finally, compared to the average GEBV across the subsets, the accuracies of the GEBV based on all variants were 0.3, 1.5, and 1.0 % lower for SCS, PY, and IFL, respectively.

**Fig. 4:**
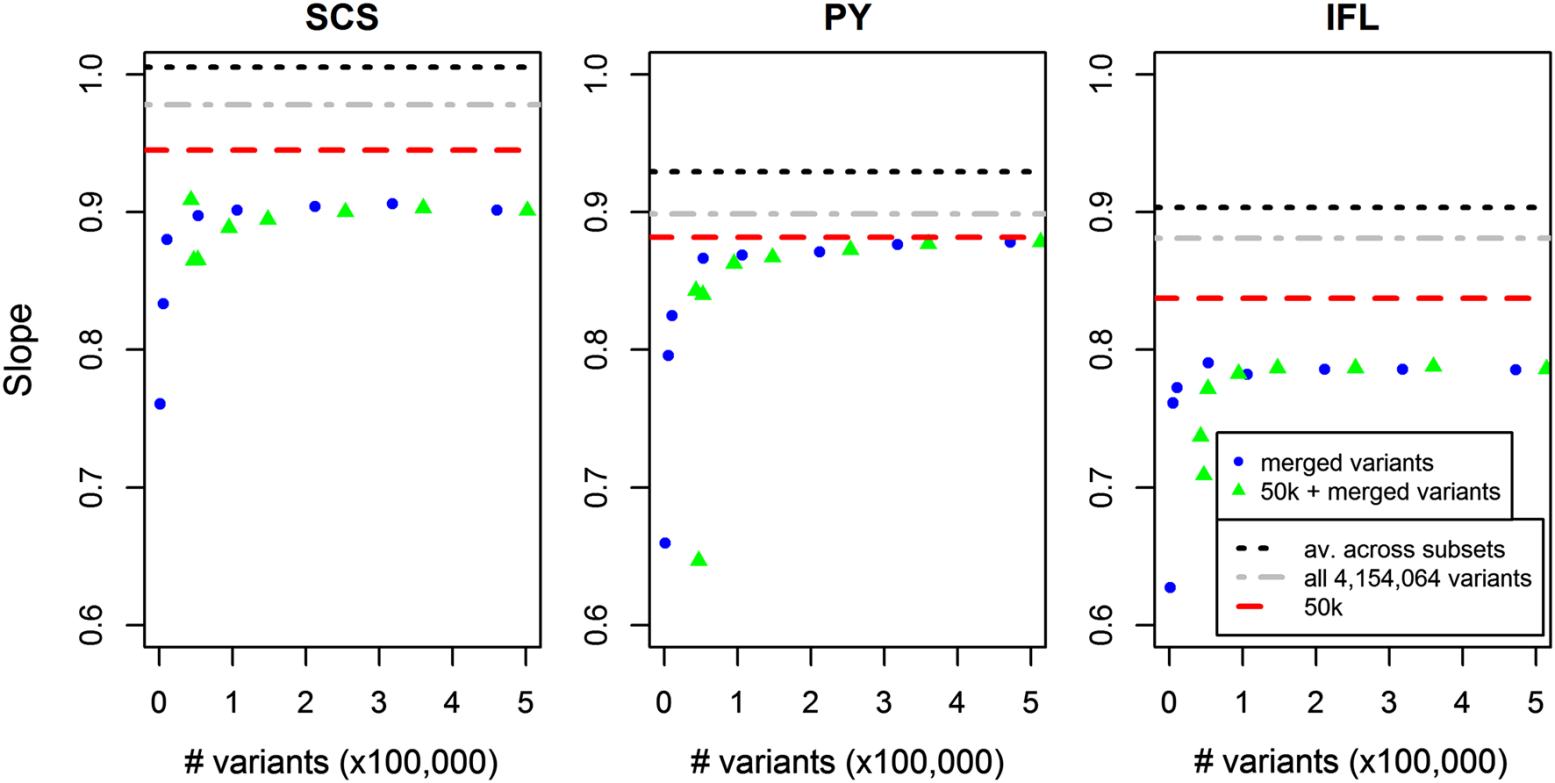
Slope of the regression of observed EBV on the GEBV when using increasingly more variants in the merged dataset for SCS, PY and IFL. Merged variants were either included alone (*blue dots*) or combined with the 41,682 50k chip SNPs (*green triangles*). *Horizontal lines* indicate the slope obtained using the average GEBV across subsets (*dashed black*), GEBV computed using all 4,154,064 variants (*dot-dashed grey*), or the 50k SNPs (*red dashed*)

Bias of the GEBV was assessed as the coefficient of the regression of observed EBV on the GEBV (Fig. 4). This showed that, in almost all cases, the bias of the GEBV based on any of the merged datasets was greater than that of the GEBV obtained with the 50k SNPs, since the regression coefficients were even more smaller than 1 than for the GEBV based on the 50k SNPs. The GEBV based on all 4,154,064 variants were less biased than those based on the 50k SNPs, while the average of the GEBV computed for each of the 106 subsets were the least biased for all three traits.

To investigate the potential impact of the value used for the parameter $\pi$, analyses based on the 50k SNPs alone or supplemented with 1060 to 53,000 sequence-based variants were repeated by setting a π value that assumed that, in each analysis, 4154 variants had a large effect. The results obtained, compared to those using a $\pi$ value of 0.999, showed that the value of $\pi$ has little effect on the accuracy and bias of the final GEBV (Table 2).

**Table 2:** Prediction accuracies and bias of the GEBV

Subset	π	Accuracy			Slope		
		SCS	PY	IFL	SCS	PY	IFL
50k	0.999	0.711	0.707	0.627	0.945	0.882	0.837
50k + 1060	0.999	0.712	0.702	0.603	0.909	0.843	0.737
50k + 5300	0.999	0.711	0.647	0.591	0.865	0.647	0.709
50k + 10,600	0.999	0.710	0.698	0.609	0.865	0.840	0.771
50k + 53,000	0.999	0.710	0.701	0.606	0.888	0.862	0.782
50k	0.900	0.714	0.703	0.625	0.953	0.879	0.839
50k + 1060	0.903	0.710	0.694	0.609	0.897	0.829	0.778
50k + 5300	0.912	0.704	0.685	0.607	0.862	0.813	0.772
50k + 10,600	0.921	0.705	0.691	0.601	0.867	0.831	0.764
50k + 53,000	0.956	0.706	0.689	0.601	0.884	0.847	0.773

GEBV are computed using 50k SNPs alone or supplemented with 1060 to 53,000 sequence-based variants. Bias is assessed as the slope of the regression of observed EBV on the GEBV

Analyses used either a π value of 0.999, or a value calculated assuming that 4154 (i.e. 0.1 % of 4,154,064) variants were assumed to have a large effect

### Effective size of Gibbs sampling chain

**Fig. 5:**
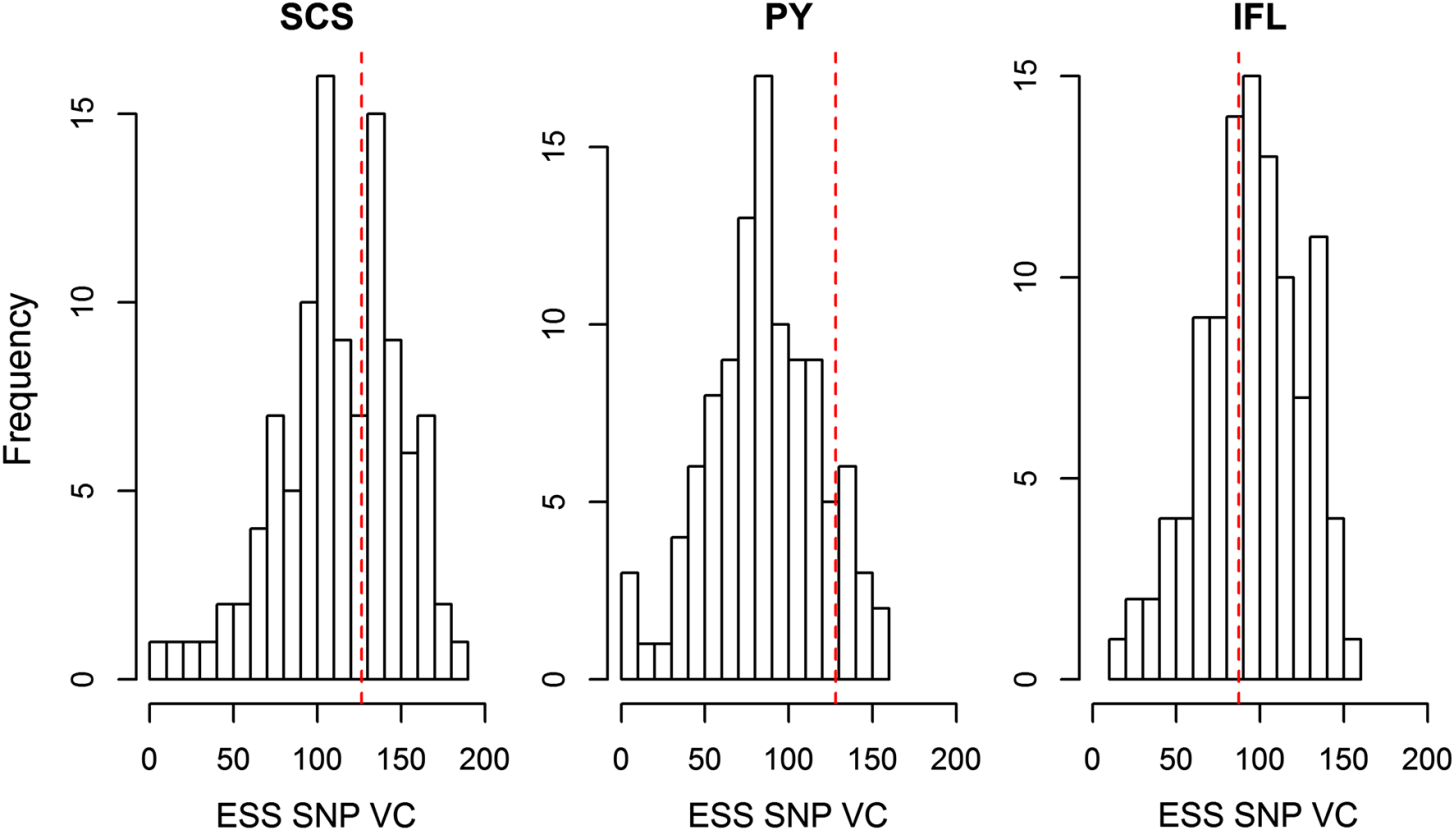
Effective sample size of the SNP variance component (ESS SNP VC) achieved for each of the 106 subsets for SCS, PY and IFL. The *dashed red vertical lines* indicate the effective sample size of the SNP variance component obtained using 41,682 SNPs included on the 50k-SNP chip and a Gibbs chain of 30,000 iterations

Effective sample sizes of the SNP variance component of all the analyses were computed to compare the model convergence. A larger effective sample size achieved with the same number of Gibbs iterations indicates better mixing of the Gibbs chains and hence better convergence. Within traits across the subsets, average effective sample sizes of 112.7, 84.8 and 94.6 were obtained, respectively, for SCS, PY and IFL (Fig. 5). Effective sample sizes obtained with the 50k SNPs, when analysed with a Gibbs chain of 30,000 iterations as for the subsets, were equal to 126.4, 128.1 and 87.2.8 for SCS, PY and IFL, respectively and were close to the average (SCS and IFL) or higher than the average value (PY) obtained across the subsets (Fig. 5).

**Fig. 6:**
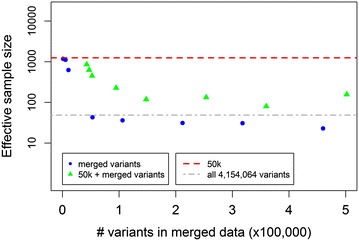
Effective sample size of the SNP variance component averaged within the computation strategy across SCS, PY and IFL. Merged variants were either included alone (*blue dots*) or combined with the 41,682 50k chip SNPs (*green triangles*). *Horizontal lines* indicate the effective sample size obtained using the 50k SNPs (*dashed red*) or all 4,154,064 variants (*dot-dashed grey*)

The effective sample size based on a Gibbs chain of 300,000 iterations with 50,000 discarded as burn in, averaged across the three traits, was equal to 1247.4 for the 50k SNPs (Fig. 6). The effective sample size obtained with the merged dataset was large regardless of whether 1060 or 5300 variants were used, but decreased rapidly as the number of included variants increased (Fig. 6). Adding the 50k SNPs to the merged datasets yielded somewhat smaller effective sample sizes with 10,600 or less variants included in the merged dataset, but larger effective sample sizes as the number of variants included in the merged dataset increased. Finally, including all 4,154,064 variants in the analysis resulted on average in an effective sample size of 48.6.

The values of these effective sample sizes show that 300,000 iterations with 50,000 discarded for burn-in was in general sufficient for the merged datasets and the data including all 4,154,064 variants. To investigate whether a smaller number of iterations would lead to the same result, in spite of the smaller effective chain sizes, GEBV for the validation animals were also computed using only 50,000 iterations after the burn-in. For the merged final datasets, the correlation between these GEBV and those computed using 250,000 iterations after the burn-in was higher than 0.998, while it was higher than 0.9999 for the analysis using all 4,154,064 variants (results not shown). This demonstrates that a Gibbs chain based on 100,000 iterations with 50,000 as burn-in was sufficient for any of these analyses.

### Ability of the model to select variants in QTL regions

**Fig. 7:**
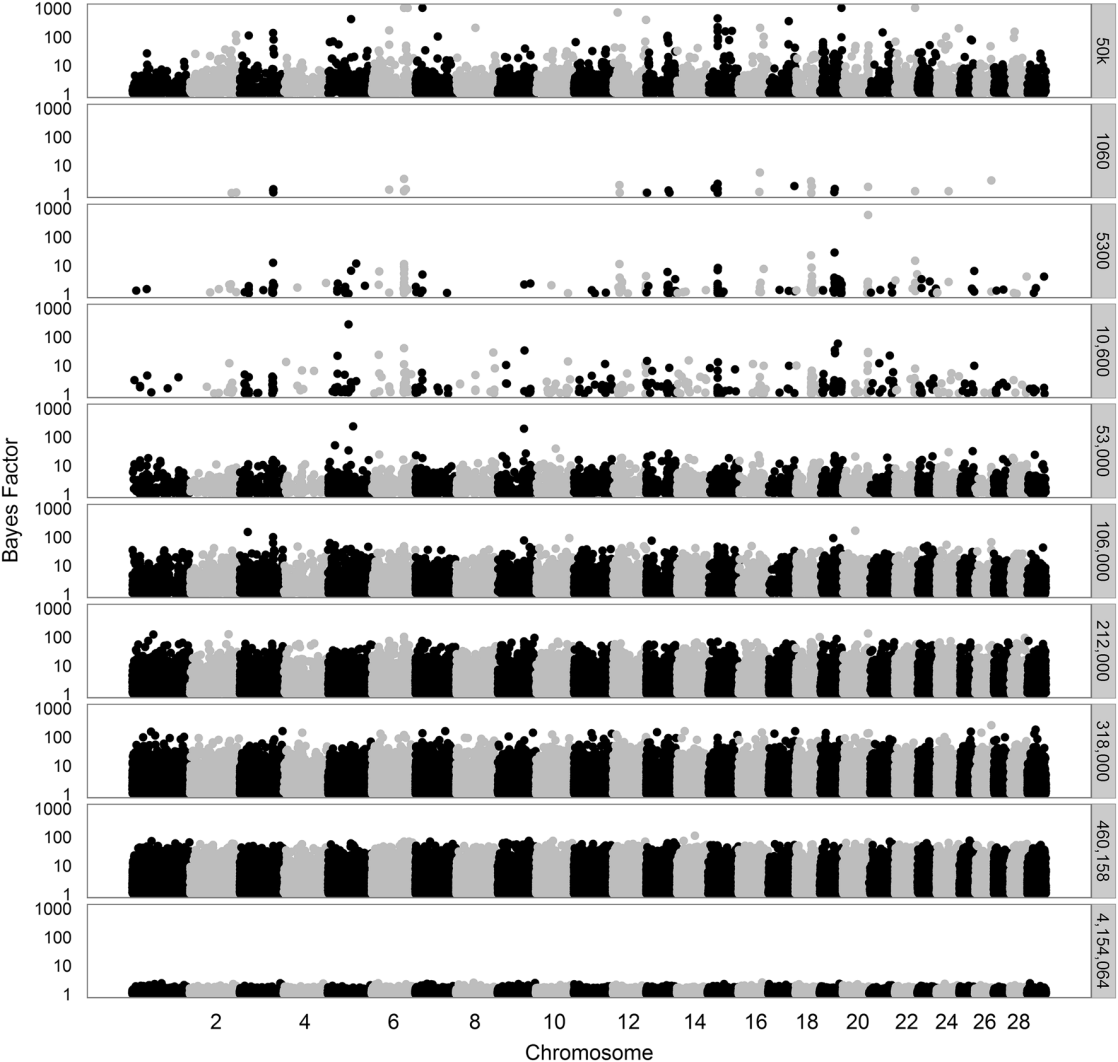
Bayes factors greater than 1 for SCS using different sets of variants. Considered sets of variants are the 50k SNPs, increasingly larger subsets (1060 to 460,158) of variants, or all 4,154,064 variants

**Fig. 8:**
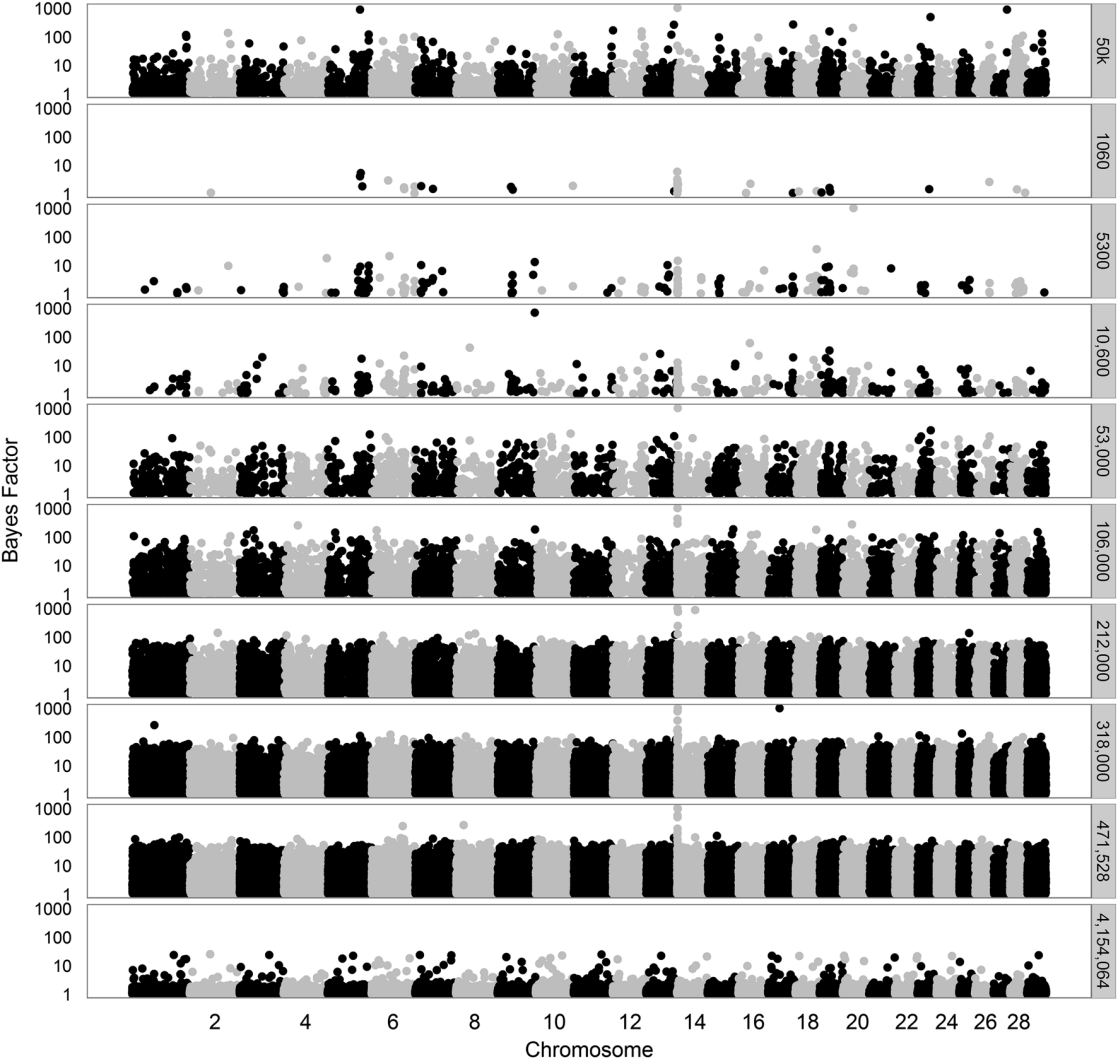
Bayes factors greater than 1 for PY using different sets of variants. Considered sets of variants are the 50k SNPs, increasingly larger subsets (1060 to 471,528) of variants, or all 4,154,064 variants

**Fig. 9:**
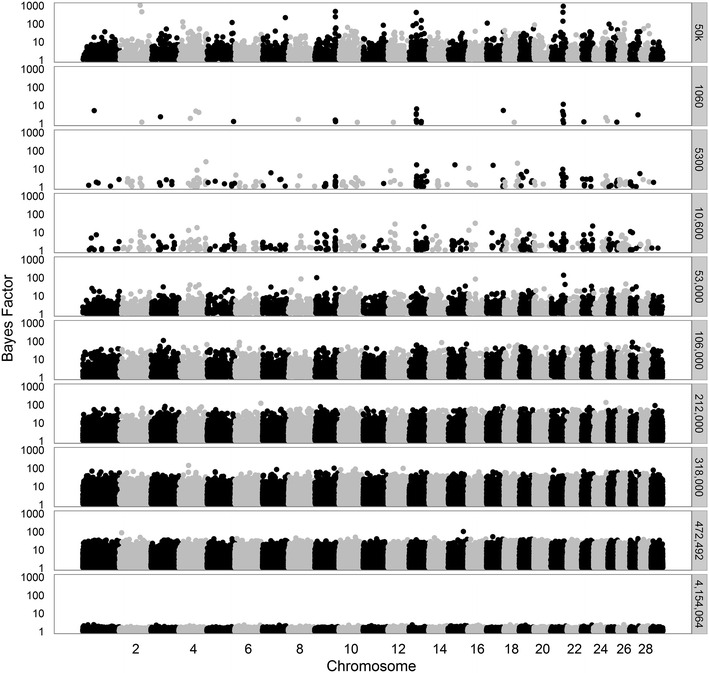
Bayes factors greater than 1 for IFL using different sets of variants. Considered sets of variants are the 50k SNPs, increasingly larger subsets (1060 to 472,492) of variants, or all 4,154,064 variants

To better understand the ability of the model to select variants in QTL regions across the different datasets, we made Manhattan plots for all Bayes factors greater than 1. First, we considered analyses based on 50k SNPs, merged data from 1060 to ~470,000 variants, or all 4,154,064 variants (Figs. 7, 8, 9, for traits SCS, PY, and IFL, respectively). The results using only the 50k SNPs showed several peaks for each of the three traits. Several of these peaks are also observed when using only the merged data including 1060 variants, although, in this case, the maximum Bayes factors were smaller. Adding more variants to the merged data, i.e. going from 1060 to ~470,000 variants, resulted in an increase of the maximum Bayes factors for some of the peaks but at a certain point, i.e. generally when more than 53,000 variants were used, most of the peaks disappeared.

Based on the prior specifications of the model, these results can be interpreted as follows. In the model, we used the same value for $\pi$ regardless of the number of variants included in the analysis. When using only 1060 variants, a proportion of 0.999 of those a priori was considered to have a “small” effect. This implies that due to this prior setting the model was hardly able to allocate a large effect to any of the variants in many iterations of the Gibbs chain. As a result, the maximum posterior probabilities and Bayes factors were relatively low in these situations. As the number of added variants increased, on average the size of the effects sampled from the distribution of “small” effects decreased, because these variants were assumed to be less strongly associated with the trait than those already included in the merged dataset. Thus, variants that displayed a strong association in more iterations of the Gibbs chain were assigned a large effect, which generally yielded larger Bayes factors than when a limited number of variants were included in addition to the initial 1060 variants. However, if the number of included variants increased considerably, the competition between variants to capture a certain effect increased and the estimated effects per variant decreased, which is translated by the model as a decrease in the amount of evidence for a variant to have a strong association with a trait. This eventually leads to a lower baseline of the Bayes factors, as was clearly the case when all variants were included.

**Fig. 10:**
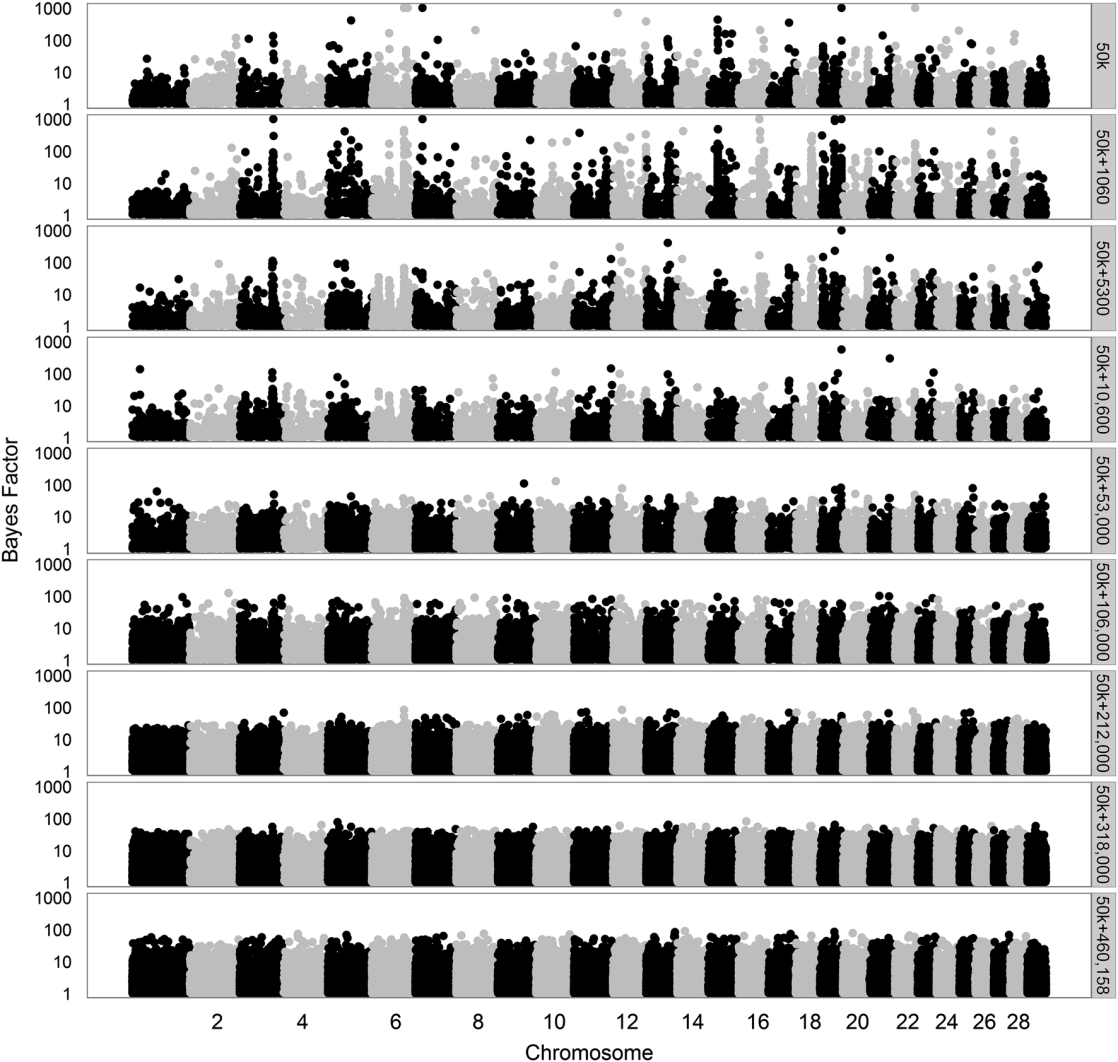
Bayes factors greater than 1 for SCS using 50k SNPs and increasingly more selected sequence-based variants. Considered sets of variants are the 50k SNPs, or the 50k SNPs plus increasingly larger subsets (1060 to 460,158) of variants

**Fig. 11:**
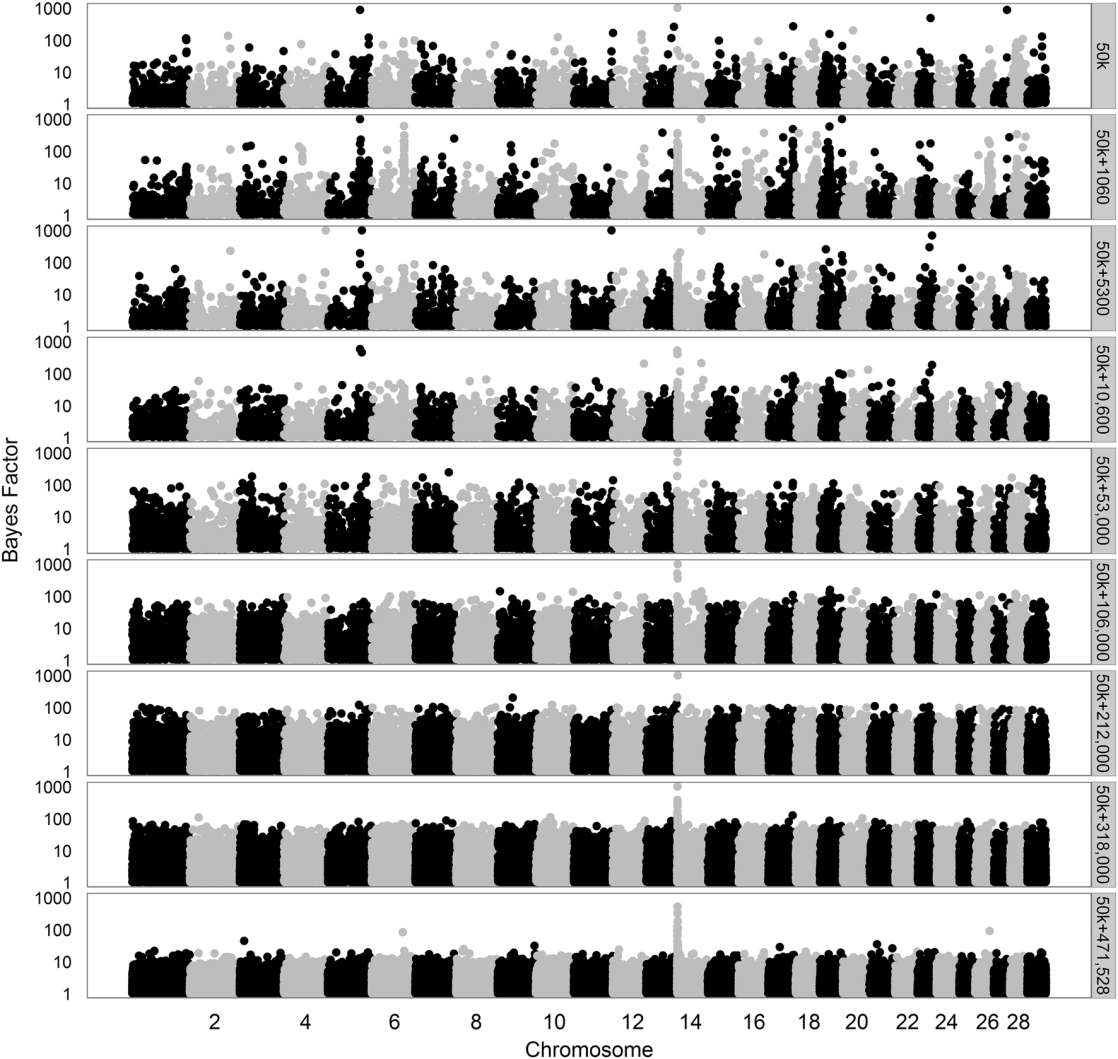
Bayes factors greater than 1 for PY using 50k SNPs and increasingly more selected sequence-based variants. Considered sets of variants are the 50k SNPs, or the 50k SNPs plus increasingly larger subsets of variants (1060 to 471,528)

**Fig. 12:**
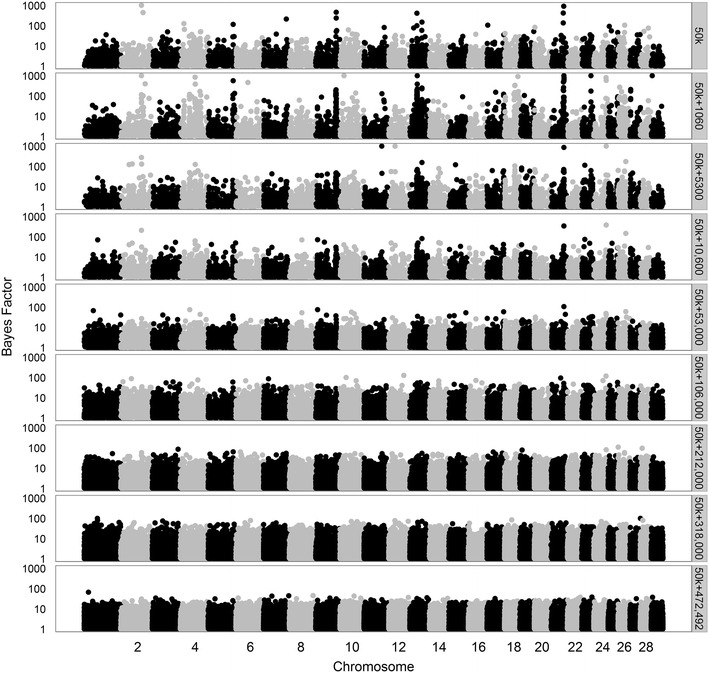
Bayes factors greater than 1 for IFL using 50k SNPs and increasingly more selected sequence-based variants. Considered sets of variants are the 50k SNPs, or the 50k SNPs plus increasingly larger subsets of variants (1060 to 472,492)

The trends in Bayes Factors that were observed when only merged datasets including a limited number of sequence-based variants were used (Figs. 7, 8, 9), changed drastically when the 50k SNPs were added in the analysis (Figs. 10, 11, 12 for traits SCS, PY, and IFL, respectively). This is because the merged dataset contained all 50k SNPs of which a large number most likely had (very) small effects. The trends in Bayes factors that were observed when only merged datasets including a large number of variants were used, were also found when these same variants and the 50k SNPs were included in the analysis, simply because the relative impact of the 50k SNPs on the results rapidly decreased as the number of sequence-based variants included increased. To further investigate the impact of increasing the number of sequence-based variants in the analysis in addition to the 50k SNPs, we zoomed in on the Manhattan plot for PY across two chromosomal regions that are known to have associations to the investigated traits: (1) the *DGAT1* region [31, 32] (see Additional file 2: Figure S2), and (2) the region that comprises the casein genes *CSN1S1*, *CSN1S2*, *CSN2*, and *CSN3* [33] (see Additional file 3: Figure S3). The results of these plots show that in the analyses based on the merged datasets, the number of loci with high Bayes factors in regions that have well-known large effects was considerably enriched, although they did not generate more precise or higher Bayes factor peaks.

Overall, in terms of Bayes factors, the clearest peaks were observed when the 50k SNPs and the 1060 sequence-based variants with the strongest association with the trait were included in the analysis.

### Computing resources

The BSSVS analysis on the subsets that comprise 3415 animals with phenotypes and on average 39,189 variants, which involved a Gibbs chain of 30,000 iterations, took ~140 min and required 108 Mb of memory. Increasing the number of iterations of the Gibbs chain naturally results in a linear increase in CPU time. Increasing the number of variants also resulted in a linear increase in CPU time and a linear increase in memory use. For instance, the BSSVS analysis for all 472,492 selected variants for IFL, which involved a Gibbs chain of 300,000 iterations, took 277 h and required a peak memory of 1.01 Gb. The BSSVS analyses including all 4,154,064 variants, which also involved a Gibbs chain of 300,000 iterations, took on average across the three traits ~110 days and required a peak memory of 9.4 Gb.

## Discussion

The objective of this study was to investigate the accuracy of SAM Bayesian variable selection applied to whole-genome sequence data. Our initial analyses showed that pruning for (near) complete LD is very important for applications of Bayesian genomic prediction models that explicitly estimate a SNP variance component using sequence data, since it may reduce the performance of these models. Based on our results, pruning within the subsets based on an LD threshold of 0.9 is recommended. Whether this threshold is sufficient can be easily monitored by checking the variance of the GEBV in the training data, where inflated values indicate impaired model performance and probably decreased prediction accuracy.

Our results showed that the SAM Bayesian variable selection model provides a procedure to consider all sequence-based variants in the prediction model, while at least part of the analysis can be performed in parallel. At the same time, the performance of the model in terms of achieved effective sample size of the estimated SNP variance component was, within the subsets, reasonably similar to that of the analysis based on the 50k SNPs. However, for the merged datasets, increasing the number of included variants required an increasingly longer Gibbs chain to achieve the same effective sample size. The accuracy achieved based on the merged data was at best similar to that based on the 50k SNPs, and only slightly larger when the average GEBV across the subsets was used to predict the validation bulls.

### (Lack of) Benefit of using sequence data

Results of simulation studies showed that the impact of using whole-genome sequence data on the accuracy of genomic prediction depends, at least partly, on the characteristics of the simulated data. Gains in accuracy were reported to be either large when causal variants had a low MAF [34] or the number of QTL was relatively small [5, 6], or small but significant when the MAF of the causal variants had a neutral distribution [34], or small to virtually non-existent when the simulated data reflected Holstein–Friesian data [35, 36]. However, recent empirical studies using whole-genome sequence data observed at best only a very small increase in accuracy compared to using the 50k or HD chips [16, 17, 37]. In our study, simply using the average GEBV across all 106 subsets yielded a marginal increase in accuracy compared to using 50k SNPs. It should be noted that using such an average GEBV is a somewhat simplistic approach. Normally, when different sources of information are for instance combined in a selection index, the covariances between those sources of information are considered. In this respect, simply taking the average GEBV is the same as assuming that the correlations between GEBV from all possible pairs of split subsets are the same, and that the accuracy of the GEBV of each of the split subsets is also the same. This assumption may be approximately valid, since LD was considered (based on MAF) when variants were allocated to different subsets.

Using all variants, either simultaneously or by computing average GEBV across all subsets, resulted in GEBV that were considerably less biased than the 50k GEBV. The GEBV of the merged subsets were the most biased i.e. had the most inflated variance. These results can be explained as follows. Selection of variants and the subsequent prediction based on the merged dataset were both based on the same phenotypic data. Variants for which effects were overestimated in the first step, are more likely to be selected into the merged dataset, than other variants. As a result, estimated variant effects and estimation errors are correlated, and the effects of the selected variants will tend to be overestimated in the second step as well [38], which results in an inflated GEBV variance. These inflated GEBV variances can probably be avoided only by performing variant selection and the final prediction in independent datasets. The 50k SNPs are selected to have a high MAF, and thus they can capture a large amount of the variance with a relatively low number of SNPs. This ascertainment bias apparently also introduces some bias in the GEBV. Finally, using average GEBV across all subsets was the approach that introduced the least bias in the GEBV.

Several reasons may explain the generally observed lack of improvement in prediction accuracy when using imputed sequence data: (1) impaired accuracy of imputed genotypes relative to e.g. 50k genotypes; (2) causal mutations may have a much lower MAF than most of the variants on commonly used SNP arrays, and may therefore be more easily filtered out of the data; and (3) imputation accuracy of sequence variants with a low MAF was shown to be, in general, poorer than that of common variants [21, 39], which would indicate that the value of such variants in the prediction process may be low. Other reasons that may explain why prediction accuracy was not improved are related to the model. One issue might be that the model fails to put sufficient weight on the causal variants due to the increasing importance of the *n* ≪ *p* problem, or because a more correct identification of the variants that have strong associations simply did not improve the prediction for the considered validation animals. These topics related to model performance will be discussed in the next section.

In our study, the validation population consisted of animals from generations after those from which the training animals originated, e.g. 84 % of the validation animals had their sire included in the training dataset. If only the 341 validation bulls without their sire in the reference population were considered, similar results (not shown) were obtained, probably because this subset of bulls still had strong relationships with the reference population. The relatively strong relationships between validation and training animals may explain why there was no benefit from including e.g. the 1060 sequence-based variants that had a strong association to the traits in addition to the 50k SNPs, although the Bayes factors suggested that this particular analysis performed slightly better to detect loci that were strongly associated with the trait of interest. It should be noted that this scenario is very similar to the approach of Brøndum et al. [13], in which 1623 QTL that had been filtered for associations with traits of interest, were added to a custom chip in addition to the 50k SNPs. These authors observed small increases in reliability up to 5 percentage points. A similar approach that consists in detecting QTL across rather than within breeds, may lead to similar or slightly greater improvements in reliability. Such applications using sequence-based variants are also expected to increase accuracies across multiple generations, and also for a breed not included in the reference population.

One of the specific aims of our application of the SAM approach, was to alleviate issues due to the severe *n* ≪ *p* problem when using whole-genome sequence data. On the one hand, our results clearly showed that reducing the number of variants based on the SAM approach resulted in a considerable increase in effective sample sizes of the Gibbs chain. On the other hand, given that the accuracies did not change when the Gibbs chain was increased from 100,000 to 300,000 samples and all 4,154,064 variants were included (results not shown), one can question the importance of the effective sample size of the SNP variance component when many variants in high LD are included in the model. In this case, there is probably a very large number of different sets of estimated variant effects that all yield very similar results in terms of GEBV.

The issue of high LD, which may extend across long distances and therefore over many variants, can be, at least partly, solved by preselecting variants. Attractive approaches to do this, are either to perform the SAM strategy proposed here, or a GWAS [40] across multiple breeds. Using multiple breeds is anticipated to yield much narrower peaks and thus much more precise identification of significant loci than within-breed analyses, because LD decays much faster across than within breeds.

In conclusion, the SAM approach applied to whole-genome sequence data did not improve prediction accuracies in our study, probably because training and prediction took place within a single breed in which relationships between animals are high. This is also in agreement with the results of MacLeod et al. [36]. Nevertheless, the SAM approach has the potential to filter important sequence-based variants that should help to improve the accuracy of across-breed or multiple-breed genomic prediction, for which results of simulation [41] and empirical studies [18] suggest that focusing on SNPs in QTL regions does lead to an improvement in accuracy of genomic prediction.

### Prior specification Bayesian variable selection model

One of the features of variable selection models is that they propose a priori variances that lead to differential shrinkage across loci. Overall, the differences between any of the prediction models belonging to the so-called “Bayesian Alphabet” type [42], are due to differences in prior specifications, which ultimately result in differences in differential shrinkage between these models [43]. Based on empirical studies that mostly used common 50k-SNP chips, such differences are not consistent and thus do not favour any of these models [43]. An unanswered question is whether differences between these models will appear when applied to whole-genome sequence data, possibly combined with the SAM or any other procedure to pre-select variants.

As the number of included variants increases, it may become necessary to adapt the model settings to the specific characteristics of the data. In the majority of the analyses, we considered a priori that 99.9 % of the variants of all the datasets had a small effect. In reality, this number may be quite different across the range of datasets considered. An alternative strategy is to assume that the absolute number of variants with a large effect remains the same regardless of the number of variants included, and to adapt $\pi$ accordingly [16]. We tested this approach for several of the merged datasets (Table 2), and concluded that the value of $\pi$ had little impact on the final GEBV. However, the value of $\pi$ does affect the estimated effects of individual loci. Assuming the same $\pi$ when many more variants are included may result in too many variants, which actually have a small effect, being allocated a large effect in the model. As a result, the size of large effects allocated by the model will decrease considerably, as observed in our study. This suggests that the optimal proportion of variants that are a priori assumed to have a large effect in the model, increases as the number of variants included increases, but at a slower rate than the number of variants itself.

When using whole-genome sequence data, the level of LD between close variants is very high. After additional editing, the number of variants in the dataset decreased by 65 % by pruning for $r^{2}$ values between genotypes of 1. The effective number of loci relative to the total number of loci is expected to decrease if the level of LD increases. Perhaps prior probabilities should be defined as the effective number of loci expected to be associated with a trait, divided by the total number of effective loci. Nevertheless, there is currently no straightforward available procedure to determine the number of loci that really affect a trait. The high level of LD could also be taken into consideration in the prior specification of the model. Although few studies have investigated this approach, one study showed that it results in small increases in accuracy of genomic prediction when the SNP density used is comparable to that of 50k-SNP chips or lower [44]. Another study showed that considering the dependency between SNPs may lead to a more parsimonious parameterization of the model [45], which may be also achieved in situations with high LD between SNPs by modelling haplotypes instead of SNPs, e.g. [46].

### Efficiency

Our results confirmed that the SAM procedure combined with Bayesian variable selection models provided a procedure that can use whole-genome sequence in genomic prediction models while limiting the overall computing time. Assuming that all analyses of the split datasets can be performed in parallel, the total computation time was 124 h, i.e. 5 h for the split analysis and 119 h for the analysis of the merged data, considering the scenario in which 212,000 selected variants were included in the final analysis. The computing time increased linearly as the number of variants included increased, and as a result the analysis of all 4,154,064 variants with a Gibbs chain of 300,000 iterations took 111 days. Processing fewer variants simply results in less computation time for a single iteration of the Gibbs chain. At the same time, our results showed that pre-selection of variants leads to faster convergence, thus to decreased length of the Gibbs chain.

## Conclusions

We showed that the SAM approach, combined with the BSSVS model, was able to perform genomic prediction efficiently using whole-genome sequence data. Effectively, it splits one large computational task into many much smaller ones, and thereby makes use of parallel processing. As a result, the whole procedure can be completed in less than two days, considering that only a few thousand variants from the sequence data are included in the final merged dataset, instead of 111 days when all sequence-based variants are included simultaneously. If the average of the GEBV across subsets is used, with parallel processing of the analyses, computation time can be shortened to 5 h. In addition, the SAM procedure improved the mixing properties of the Gibbs chains in the analysis, simply by reducing the total number of variants included in any of the analyses. In particular, it is important to prune variants, within the subsets, that have complete or high levels of LD, i.e. for which $r^{2}$ values between genotypes are greater than 0.9 since they reduce the prediction performance. Predictions based on variants in the merged dataset did not outperform those based on the 50k-SNP chip, although we observed that the model was able to considerably enrich the number of selected variants in a few well-known QTL regions, even when only small to moderate numbers of selected sequence-based variants were added. One drawback of the SAM procedure is that it leads to bias in terms of inflated GEBV variances, which, probably, can be avoided only by performing variant selection and the final prediction in independent datasets, a procedure that may be difficult to perform in practice. Using GEBV computed as the average of the GEBV computed in each of the subsets, achieved the highest accuracy for all three traits, i.e. it was 0.5 to 1.1 % higher than the accuracies obtained with the 50k-SNP chip. These average GEBV were also the least biased for all three traits analyzed. Lack of improvement in prediction accuracy with the SAM approach in our study was probably due to training and prediction being performed within a single breed with high relationships between individuals. Nevertheless, the SAM approach may have the potential to improve accuracy of across-breed or multiple-breed genomic prediction.

## Additional files

10.1186/s12711-016-0225-x Accuracy versus variance of the realized GEBV for SCS in subsets that required additional LD pruning (A), and accuracies after versus before pruning for the same subsets (B).
10.1186/s12711-016-0225-x Bayes factors greater than 1 for PY using 50k SNPs and increasingly larger sets of selected variants in the *DGAT1* region. Considered sets of variants are the 50k SNPs, or the 50k SNPs plus increasingly larger subsets of variants (1060 to 471,528). Bayes factors are plotted for 1 to 3 Mbp on chromosome 14. The red vertical lines indicate the position of the *DGAT1* gene.
10.1186/s12711-016-0225-x Bayes factors greater than 1 for PY using 50k SNPs and increasingly larger sets of selected variants in the region of the casein genes *CSN1S1*, *CSN1S2*, *CSN2*, and *CSN3*. Considered sets of variants are the 50k SNPs, or the 50k SNPs plus increasingly larger subsets of variants (1060 to 471,528). Bayes factors are plotted for 85 to 89.5 Mbp on chromosome 6. The green, red, blue and orange vertical lines indicate, respectively, the position of the *CSN1S1*, *CSN2*, *CSN1S2*, and *CSN3* genes.
