# Supplementary figures and images for: Efficient genomic prediction based on whole-genome sequence data using split-and-merge Bayesian variable selection
Source: Genet Sel Evol. 2016 Jun 29;48:49. doi: 10.1186/s12711-016-0225-x (PMC4926307; doi:10.1186/s12711-016-0225-x)

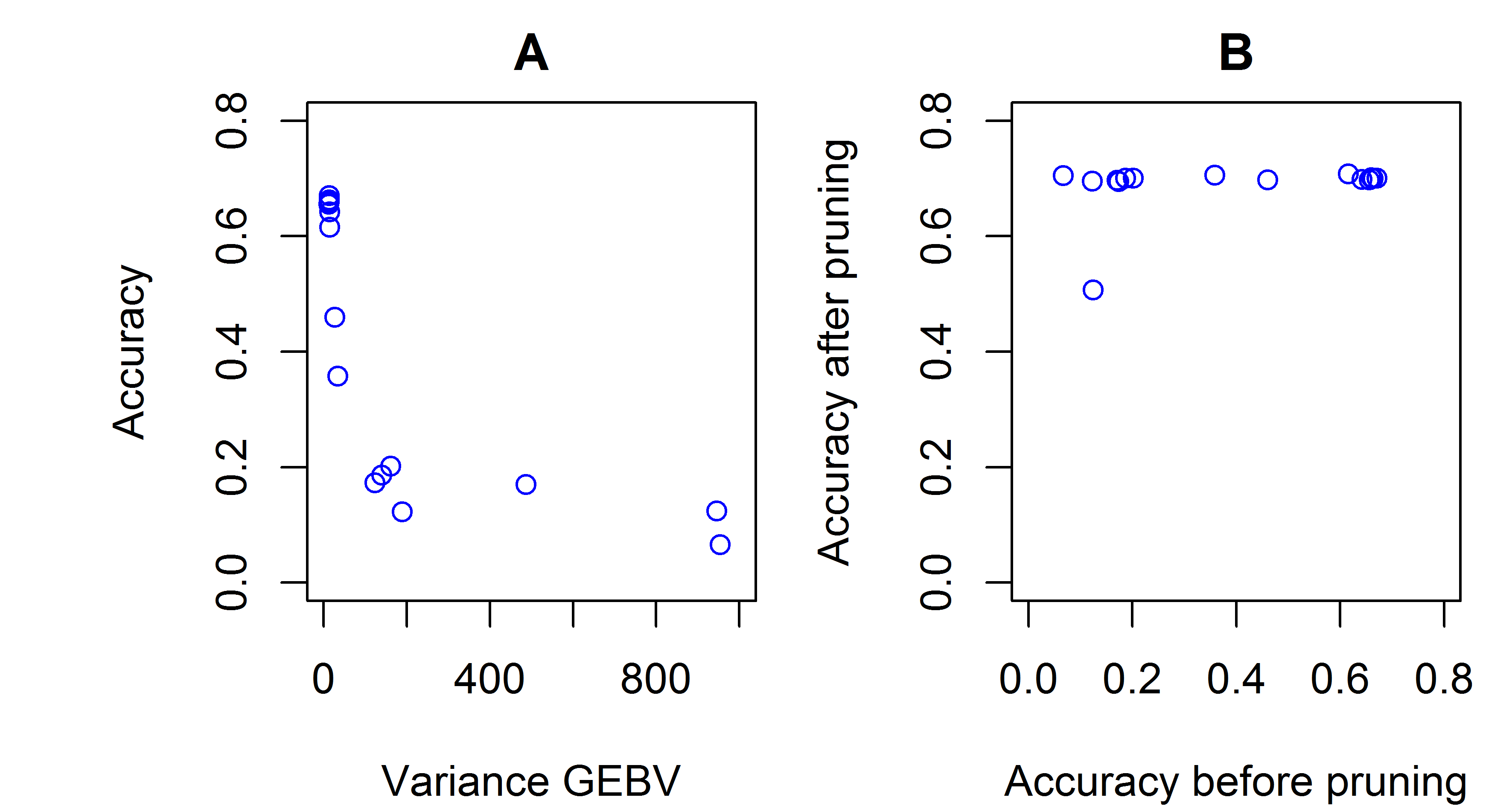

Supplement: Supplementary file 1 — 10.1186/s12711-016-0225-x Accuracy versus variance of the realized GEBV for SCS in subsets that required additional LD pruning (A), and accuracies after versus before pruning for the same subsets (B). [file 12711_2016_225_MOESM1_ESM.png]

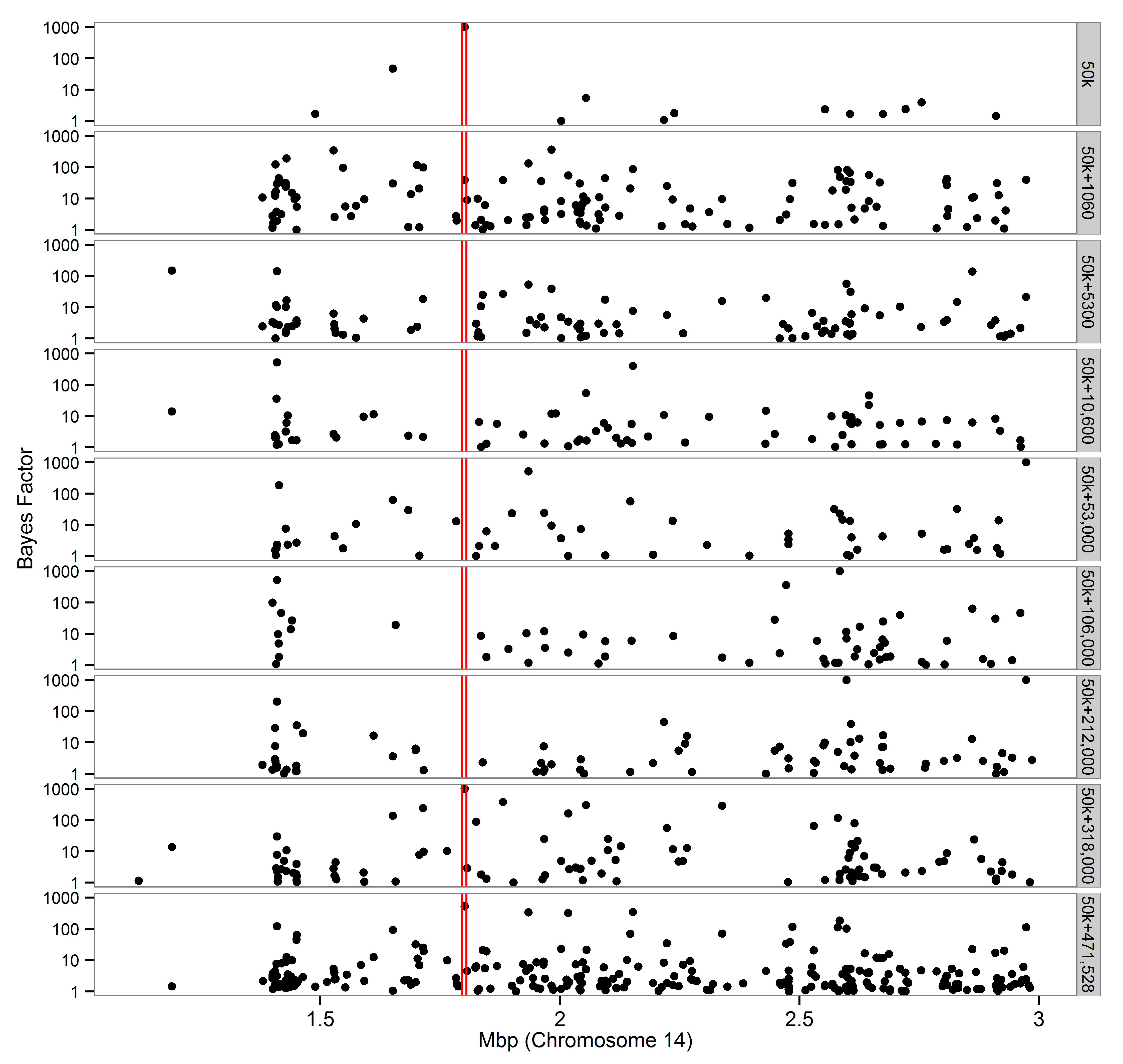

Supplement: Supplementary file 2 — 10.1186/s12711-016-0225-x Bayes factors greater than 1 for PY using 50k SNPs and increasingly larger sets of selected variants in the DGAT1 region. Considered sets of variants are the 50k SNPs, or the 50k SNPs plus increasingly larger subsets of variants (1060 to 471,528). Bayes factors are plotted for 1 to 3 Mbp on chromosome 14. The red vertical lines indicate the position of the DGAT1 gene. [file 12711_2016_225_MOESM2_ESM.png]

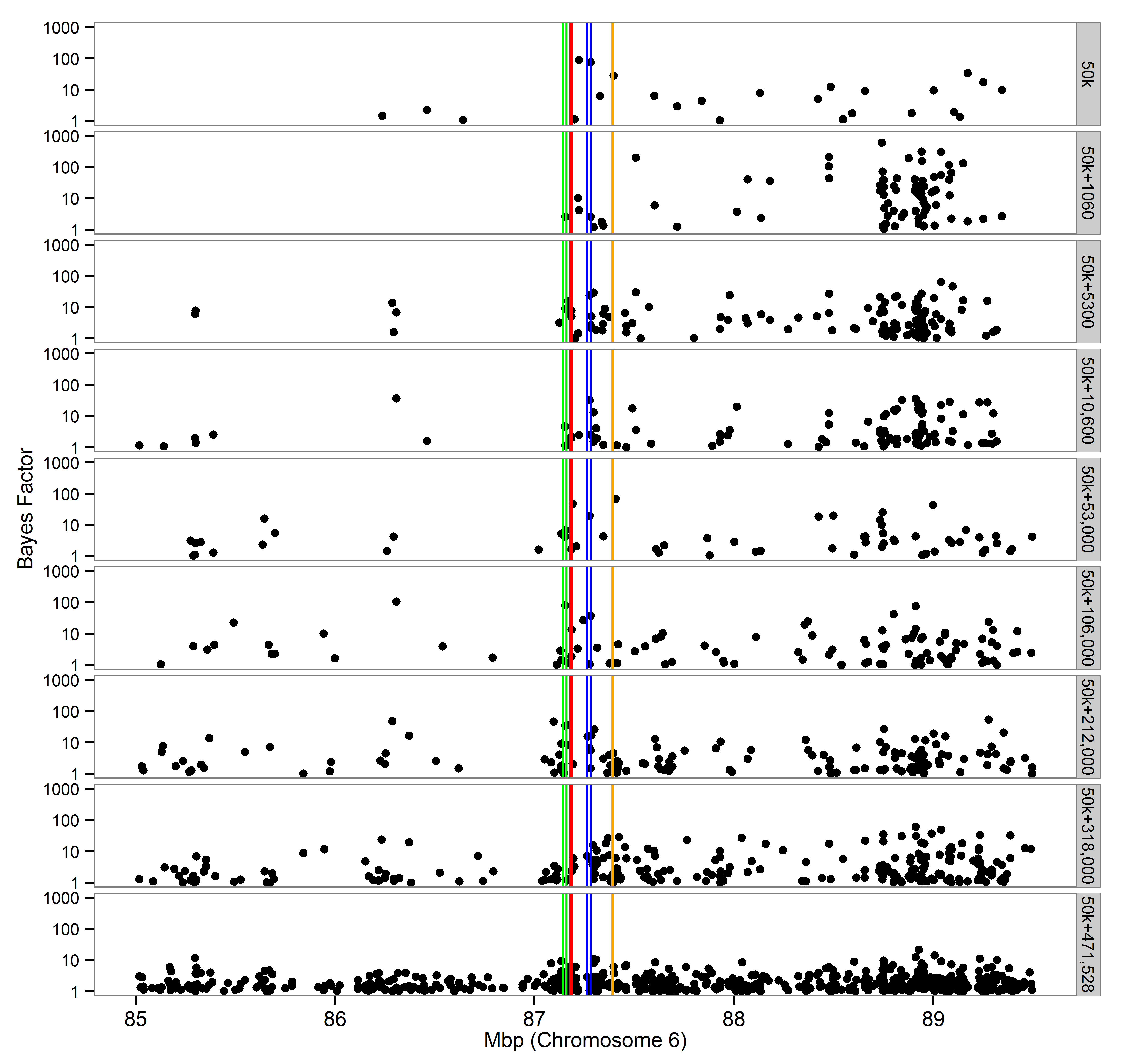

Supplement: Supplementary file 3 — 10.1186/s12711-016-0225-x Bayes factors greater than 1 for PY using 50k SNPs and increasingly larger sets of selected variants in the region of the casein genes CSN1S1, CSN1S2, CSN2, and CSN3. Considered sets of variants are the 50k SNPs, or the 50k SNPs plus increasingly larger subsets of variants (1060 to 471,528). Bayes factors are plotted for 85 to 89.5 Mbp on chromosome 6. The green, red, blue and orange vertical lines indicate, respectively, the position of the CSN1S1, CSN2, CSN1S2, and CSN3 genes. [file 12711_2016_225_MOESM3_ESM.png]
